# Age, Sex Hormones, and Circadian Rhythm Regulate the Expression of Amyloid-Beta Scavengers at the Choroid Plexus

**DOI:** 10.3390/ijms21186813

**Published:** 2020-09-17

**Authors:** Ana C. Duarte, André Furtado, Mariya V. Hrynchak, Ana R. Costa, Daniela Talhada, Isabel Gonçalves, Manuel C. Lemos, Telma Quintela, Cecília R.A. Santos

**Affiliations:** 1 CICS-UBI—Health Sciences Research Centre, University of Beira Interior, Av. Infante D. Henrique, 6200-506 Covilhã, Portugal; anacduarte28@hotmail.com (A.C.D.); andre.furtado@ubi.pt (A.F.); anarfcosta1990@gmail.com (A.R.C.); dtalhada@gmail.com (D.T.); igoncalves@fcsaude.ubi.pt (I.G.); mclemos@fcsaude.ubi.pt (M.C.L.); csantos@fcsaude.ubi.pt (C.R.A.S.); 2 Department of Neurobiology, School of Biology/Chemistry, University of Osnabrück, 49074 Osnabrück, Germany; marya.hrynchak@gmail.com

**Keywords:** choroid plexus, amyloid-beta scavengers, blood–cerebrospinal fluid barrier, age, sex hormones, circadian rhythm

## Abstract

Accumulation of amyloid-beta (Aβ) in the brain is thought to derive from the impairment of Aβ clearance mechanisms rather than from its overproduction, which consequently contributes to the development of Alzheimer’s disease. The choroid plexus epithelial cells constitute an important clearance route for Aβ, either by facilitating its transport from the cerebrospinal fluid to the blood, or by synthesizing and secreting various proteins involved in Aβ degradation. Impaired choroid plexus synthesis, secretion, and transport of these Aβ-metabolizing enzymes have been therefore associated with the disruption of Aβ homeostasis and amyloid load. Factors such as aging, female gender, and circadian rhythm disturbances are related to the decline of choroid plexus functions that may be involved in the modulation of Aβ-clearance mechanisms. In this study, we investigated the impact of age, sex hormones, and circadian rhythm on the expression of Aβ scavengers such as apolipoprotein J, gelsolin, and transthyretin at the rat choroid plexus. Our results demonstrated that mRNA expression and both intracellular and secreted protein levels of the studied Aβ scavengers are age-, sex-, and circadian-dependent. These data suggest that the Aβ-degradation and clearance pathways at the choroid plexus, mediated by the presence of Aβ scavengers, might be compromised as a consequence of aging and circadian disturbances. These are important findings that enhance the understanding of Aβ-clearance-regulating mechanisms at the blood–cerebrospinal fluid barrier.

## 1. Introduction

The choroid plexus (CP) epithelium is a multifunctional tissue responsible for a wide range of biochemical and cellular homeostatic actions in the central nervous system. The CP exerts important functions that include the production and secretion of cerebrospinal fluid (CSF) and of several bioactive molecules, as well as the establishment of the blood–CSF barrier (BCSFB) [1]. Considerable attention has also been given to the role of CP in the clearance of amyloid-beta (Aβ), contributing to the equilibrium of Aβ levels in the brain [2,3]. In fact, Aβ accumulation in the brain of Alzheimer’s disease (AD) patients, as well as in the CP epithelium itself [4,5], might be a result of an overproduction, inadequate metabolic clearance, or an inappropriate transport of this peptide through the BCSFB [2]. The accumulation of amyloid-like inclusions in the CP during aging is associated with several changes in CP morphology and function, of which the most prominent are decreased CSF production and turnover, changes in the metabolic activity, and diminished clearance of Aβ peptides [6,7].

As a key element in Aβ clearance routes, the CP maintains Aβ levels not only with the renewal of the CSF but also with the active secretion of several proteins involved in Aβ transport/degradation, such as apolipoprotein J (APOJ), gelsolin (GLS), and transthyretin (TTR) (reviewed in [8]). APOJ may bind, and gradually reduce, Aβ accumulation and aggregation, reducing amyloid fibrillogenesis and improving its clearance. GLS, besides the potential role described for APOJ, is also involved in neuronal survival and protection of neurons against Aβ toxicity. Particularly, in the CP, GLS is able to protect Aβ-induced cytoskeletal modifications [9]. Finally, TTR is able to bind Aβ preventing its aggregation and toxicity, but if sequestration fails, TTR is also capable of disrupting Aβ fibrils [8]. Considering all the above, once synthesized, these Aβ-related molecules are secreted into the CSF where they can form stable complexes with the Aβ peptide and hydrolyze it into less-neurotoxic fragments. Therefore, their actions delay the formation and deposition of amyloid fibrils and inhibit the cytotoxic effects induced by the Aβ peptide [10].

Interestingly, Aβ clearance seems to occur with a circadian rhythmicity, and is greatly enhanced during sleep [11]. In fact, in mice transgenic for amyloid precursor protein, Aβ levels in the interstitial fluid oscillate in a circadian manner, increasing during wakefulness (dark period) and decreasing during sleep (light period). When these animals were subjected to chronic sleep deprivation, a greater deposition of Aβ plaques occurred compared to their age-matched litter-mate controls [12]. Concurrently, a clear evidence of CSF diurnal fluctuation of Aβ was detected in young healthy male volunteers [12], and an attenuation of the circadian pattern of Aβ was observed in humans with mutations that cause autosomal-dominant AD [13]. Considering that the CP is a crucial component and regulator of the circadian clock system [14,15], the disruption of the molecular clock might lead to deficient Aβ clearance in the BCSFB. In addition to disruption of the circadian rhythm, aging and female gender are also factors that might be responsible for an insufficient removal of Aβ from the brain. Curiously, the sex hormone background causes changes in several CP pathways such as the circadian rhythm signaling [15,16], but we do not know how this affects Aβ clearance mechanisms in the CP.

In this scenario, we investigated if the expression of some genes/proteins involved in the transport/degradation of Aβ in the rat CP undergoes changes with age, sex, and circadian timing. Understanding the expression regulation of Aβ scavengers in the CP epithelium might disclose novel targets to control and/or prevent potential neurotoxic accumulation in the brain.

## 2. Results

### 2.1. Effects of Age on the Expression of APOJ, GLS, and TTR in the Choroid Plexus

To assess the effects of age on the Aβ scavengers in the rat CP tissue, we compared mRNA expression and intracellular and secreted protein levels of APOJ, GLS, and TTR in different age groups—newborn (5–7 days), young (1 month), and adult (3 months).

**Figure 1:**
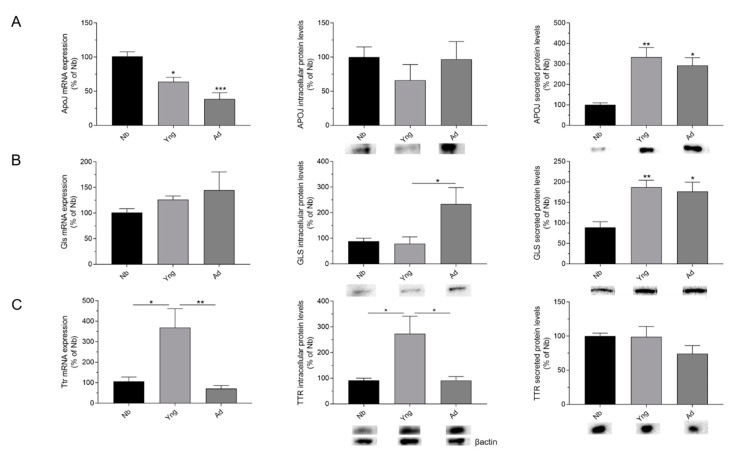
Effects of age on the expression of apolipoprotein J (APOJ), gelsolin (GLS), and transthyretin (TTR) in the choroid plexus (CP). mRNA expression, intracellular protein and secreted protein levels of APOJ (**A**), GLS (**B**), and TTR (**C**) in CP explants and conditioned media of newborn (Nb), young (Yng), and adult (Ad) rats was analyzed by real time RT-PCR and Western blot, respectively. Bar graphs represent mean ± SEM (N ≥ 3; * *p* < 0.05, ** *p* < 0.01, *** *p* < 0.001; one-way ANOVA followed by Bonferroni’s post hoc test).

We found that ApoJ mRNA expression decreased significantly with age; a significant lower expression was seen in young and adult groups compared with the newborn group (*p* < 0.05 and *p* < 0.001, respectively; Figure 1A). In addition, although intracellular APOJ protein levels remained unchanged (Figure 1A), APOJ content in the conditioned media increased in young and adult groups compared to the newborn group (*p* < 0.01 and *p* < 0.05, respectively; Figure 1A).

Regarding Gls mRNA expression, we did not find a statistically significant difference between the age groups (Figure 1B). In contrast, we observed an increase of intracellular GLS protein levels in the adult group when compared to newborn and young groups (*p* < 0.05; Figure 1B). Furthermore, an increase in secreted GLS levels was found not only in adult but also in young groups when compared to the newborn group (*p* < 0.05 and *p* < 0.01, respectively; Figure 1B).

Finally, the higher levels of Ttr mRNA expression were observed in the young group (Figure 1C; *p* < 0.05, compared to the newborn group), and declined thereafter when compared to the adult group (Figure 1C; *p* < 0.01). Likewise, the results obtained for intracellular TTR protein levels exhibited a similar pattern for the mRNA expression, showing higher levels in the young group than in the newborn group (*p* < 0.05; Figure 1C), but lower in the adult group compared to the young group (*p* < 0.05; Figure 1C). In contrast, there were no significant differences in secreted TTR protein content between the age groups (Figure 1C).

Generally speaking, effects of age were reported in mRNA expression and in cellular and secreted protein levels mainly between newborn and the young and adult groups.

### 2.2. Effects of Sex on the Expression of APOJ, GLS, and TTR in the Choroid Plexus

**Figure 2:**
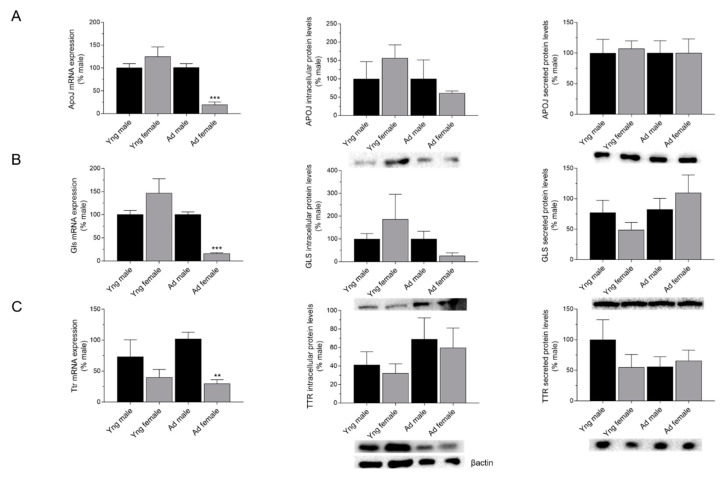
Effects of sex on the expression of apolipoprotein J (APOJ), gelsolin (GLS), and transthyretin (TTR) in the choroid plexus (CP). mRNA expression, intracellular protein, and secreted protein levels of APOJ (**A**), GLS (**B**), and TTR (**C**) in CP explants and conditioned media of male and female from young (Yng) and adult (Ad) groups was analyzed by real time RT-PCR and Western blot, respectively. Bar graphs represent mean ± SEM (N ≥ 3; ** *p* < 0.01, *** *p* < 0.001; One-way ANOVA followed by Bonferroni’s post hoc test).

To further investigate the effects of sex differences in Aβ scavengers, we compared mRNA expression and cellular and secreted protein levels of APOJ, GLS, and TTR in the rat CP between males and females from the young and adult groups. Interestingly, when the mRNA expression of ApoJ, Gls, and Ttr was examined between sexes, the expression was significantly lower in adult females when compared to adult males (*p* < 0.001 for ApoJ and Gls and *p* < 0.01 for Ttr; Figure 2A–C).

On the contrary, no significant differences in either intracellular or secreted APOJ, GLS, and TTR protein levels were observed between males and females from the young and adult groups (Figure 2A–C). In sum, sex effects were only observed in the mRNA expression of adult animals, with females showing a lower expression than males.

### 2.3. Influence of Circadian Rhythm in ApoJ, Gls, and Ttr Expression Levels

**Figure 3:**
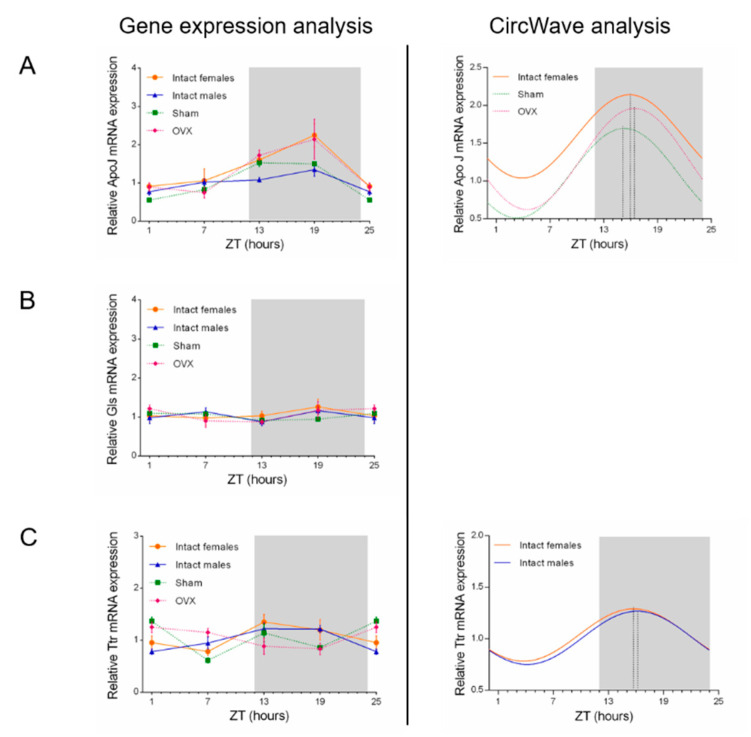
Circadian profile of choroid plexus (CP) Aβ scavengers. ApoJ, Gls, and Ttr expression was analyzed every 6 h during a 24-h period in intact females (orange), intact males (blue), sham-operated (sham; green), and ovariectomized animals (OVX, pink). Graphs represent the relative mRNA expression levels of ApoJ (**A**), Gls (**B**), and Ttr (**C**) in the CP. Each data point represents the mean ± SEM (n = 4–5). CircWave analysis (right side of the panel) shows the corresponding statistical analysis for rhythmicity (statistical analysis is shown in Table 1). No curve could be fitted for Gls circadian expression. The center of gravity (phase) is represented by the vertical line intersecting each curve. White and grey backgrounds represent light and dark phases, respectively. Data from ZT1 and ZT25 are double-plotted.

The effects of circadian rhythm in Aβ scavengers were studied in the CP of intact male and female, sham, and ovariectomized (OVX) rats. ApoJ, Gls, and Ttr expression was compared by real time RT-PCR every 6 h during a 24-h period. Among the Aβ scavengers analyzed, ApoJ and Ttr oscillated in a distinct rhythmic pattern across the 24-h cycle, whereas Gls did not show a rhythmic expression (Figure 3).

In particular, ApoJ showed a rhythmic pattern of expression in intact females, sham, and OVX animals, reaching its peak during the dark phase (ZT15-ZT16; *p* < 0.05; Figure 3A). Interestingly, the peak time (expressed as center of gravity) in the OVX group profile was not altered when compared with intact female and sham group profiles (Figure 3A). ApoJ mRNA was not rhythmically transcribed in the CP of intact males (Figure 3A).

For Gls, we found no circadian changes in mRNA expression in CP epithelium along the day, in the different groups (Figure 3B).

Finally, Ttr mRNA expression showed circadian rhythmicity in intact female and male groups (*p* < 0.05 and *p* < 0.001, respectively; Figure 3C), but not in sham and OVX animals. In intact female and male groups, Ttr peaked at approximately the same time, around ZT16 (Figure 3C). CircWave data analysis is shown in Table 1. In summary, a significant circadian rhythm was observed in ApoJ and Ttr mRNA expression in the CP epithelium, with intact females showing a circadian fluctuation in both Aβ scavengers. Gls did not display a circadian pattern.

## 3. Discussion

The aim of this study was to evaluate the influence of aging, sex and circadian rhythm, in the expression of Aβ scavengers in the CP. We found that these factors which may contribute to Aβ scavengers’ modulation, affect the expression of APOJ, GLS and TTR gene/protein levels in rat CPs depending on age, sex and daytime. We focused our study on APOJ, GLS and TTR because these Aβ related molecules are known to be synthesized by the CP epithelial cells and secreted into the CSF, contributing to the Aβ clearance route.

In particular, from our results, it is clear that ApoJ expression in CP tissue is age-, sex- and circadian-dependent. We demonstrated that ApoJ mRNA expression declines with aging and is reduced in adult females when compared to adult males. The association of normal aging with a depletion of sex hormones may support these results [17]. The literature review shows that in females, estradiol is high in newborn and in 2–4 weeks old rats. Afterwards, estradiol levels drop and begin to fluctuate every 4 to 5 days. On the other hand, testosterone levels in the male rats are elevated in the first 3–4 weeks, decline during the prepubertal period, and increase again during puberty [18,19]. Despite the limited number of studies addressing the effects of sex hormones in ApoJ levels, the evidence that lower levels of ApoJ in the CP tissue correlate with a decline of female sex hormones, is consistent with previous studies, showing an up-regulation of ApoJ expression by estrogens in endometrial cancer cell lines [20,21]. Furthermore, the decrease in mRNA expression can also be linked to the reported age-associated changes at the CP, namely the reduction of its secretory activity [22], affecting the synthesis and secretion of ApoJ. The reduction of ApoJ mRNA in adult females in comparison to males are in line with recent research demonstrating that ApoJ expression is significantly diminished in the early aging of the female brain but not in the male brain [23]. Importantly, completely opposite to the ApoJ mRNA expression trend is the increase of secreted APOJ protein levels with aging in young and adult groups. The divergent expression of protein and mRNA levels was also previously reported by other groups [24,25,26,27], and is a reflection of the mechanisms which occur after mRNA processing, such as post-transcriptional, translational and protein degradation regulation. Thus, although the effects of age and sex hormones in the expression of ApoJ mRNA/protein in CP have been clarified, the relevance of high levels of APOJ in modulating Aβ aggregation and/or clearance is still debatable. Furthermore, the presence of both a heat shock transcription factor-1 and an activator protein-element [28], besides the existence of cytosinephosphate-guanosine (CpG) islands [29] in the APOJ gene promoter, indicates a transcriptional control by regulation elements and epigenetic mechanisms, respectively. Regarding the influence of circadian rhythm on the expression of Aβ scavengers, we observed that ApoJ was rhythmically transcribed in intact females, sham and OVX rats. The data show that anaesthetic agents (in the sham group) and hormonal depletion (in the OVX group) do not affect ApoJ circadian pattern. Despite the effect of anaesthetic agents on the shifting of the circadian clock described by others [30], our results show that the combination of medetomidine and ketamine did not influence the expression pattern of ApoJ. Equally relevant is the fact that the circadian pattern of ApoJ was not disrupted by the depletion of female sex hormones during the 24-h cycle, emphasizing that the link between the circadian rhythm and sex hormones cannot be ignored in Aβ clearance mechanisms. Contrary to our expectations, ApoJ peak expression levels occurred during the night (when rats tend to be awake) and do not match our first supposition that high levels of Aβ scavengers in CP would correlate favourably with the low concentrations of Aβ in rat brain interstitial fluid during sleep (light period) [31]. Considering all the data as a whole, ApoJ expression in the CP, at both mRNA and protein levels was altered as a consequence of age, sex and circadian rhythm, although the exact contribution of these variants to the Aβ clearance mechanisms across BCSFB remained unclear.

A further novel finding of our study was an increase in intracellular/secreted GLS levels with aging. Unlike other research showing no differences of GLS protein levels in the ventral hippocampus between young and adult female rats [32], we found an increase in cellular GLS protein levels between young and adult groups. In addition, like ApoJ mRNA levels, Gls expression was lower in the CP of adult females compared to adult males. This could be explained by the drop of female sex hormones and the dysfunction of the CP along aging. In this way, the enhancement of GLS cellular protein/secreted levels in CP epithelium may represent a route to affect amyloid dynamics in the CSF. Finally, we found that Gls does not exhibit a significant circadian pattern, with the mRNA expression levels remaining relatively constant across the 24 h. Our results suggest that the constitutive expression of Gls in the CP epithelium along the day might not be directly involved in the diurnal fluctuation of Aβ clearance in the CSF.

Regarding TTR, mRNA and intracellular protein levels reached maximum values in young rats, and decreased thereafter in adult groups, validating previous reports of an age-dependent TTR decrease in the CSF [33,34]. Since TTR synthesis in the brain occurs exclusively in the CP, and is the principal protein synthesized and secreted into the CSF by the CP, TTR decrease with aging seems to be a result of CP (dys)function (reviewed by [6]). Importantly, as early demonstrated by our group, TTR is up-regulated in the CP by estradiol [35], progesterone [36] and dihydrotestosterone [37]. Besides, a putative distal estrogen response element (ERE; aAGTCAAAGTGACCa) was identified in the 5′-flanking region of hTTR gene, and demonstrated that estrogen receptors may mediate estradiol-induced expression of TTR through binding to the ERE consensus [38]. 

Finally, our research revealed a Ttr significant circadian pattern only in intact males and females. The absence of significance in sham and OVX animals anticipates an involvement of anaesthesia and/or female sex hormones in the modulation of Ttr expression levels along the day, and consequently in the regulation of Aβ clearance. As occurred with ApoJ, Ttr expression levels peaked during the night (active period) in both intact male and female animals. The recognized influence of TTR in the promotion of Aβ binding/degradation in the brain [39], suggests that the observed 24-h circadian pattern of expression in the CP promotes the clearance of Aβ during the active period (nocturnal clearance), and not during sleep as previously anticipated. These results, although contradictory at first sight, strengthened the evidence for the presence of both a dependent- and independent-sleep mechanism of Aβ clearance in the brain [40]. It was previously reported that Aβ levels in mice interstitial fluid exhibited a diurnal oscillation and the clearance of brain Aβ levels increases during sleep, underlying a regulation of the glymphatic system during that period (reviewed by [31]). Thus, the glymphatic flow suggested that Aβ diurnal fluctuations are a sleep-dependent mechanism however, independent of the circadian clock. Presumably, the influence of the circadian clock in the circadian pattern of CP’s Aβ scavengers may be assumed as a sleep- independent process that will increase the clearance of Aβ at the BCSFB. 

In summary, we were able to confirm the impact of age, sex and circadian rhythm on the regulation of Aβ scavengers’ levels. As the choroidal epithelium at the BCSFB is an important clearance route for Aβ, the modulation of the synthesis and secretion of Aβ scavengers’ that will degrade/bind Aβ, will be partly responsible for an improvement of Aβ homeostasis and amyloid load. 

## 4. Materials and Methods

### 4.1. Animals

In vivo experiments and procedures were approved and performed in compliance with the national and European Union rules for the care and handling of laboratory animals (Directive 2010/63/EU). Experiments were also carried out according to the Portuguese law for animal welfare and the protocol was approved by the Committee on the Ethics of Animal Experiments of the Health Sciences Research Centre of the University of Beira Interior (DGAV-0421/000/000/2018_008668, 4 September 2018). Newborn (5–7 days), young male and female (1 month of age), and adult male and female (3 months of age) Wistar Han rats were used to evaluate the effects of age and sex-hormone background on the expression of Aβ scavengers. Intact male and female, sham-operated, and ovariectomized (2 months ± 2 weeks of age) Wistar Han rats were used to monitor the circadian expression of Aβ scavengers over 24-h. Animals were grouped by sex and housed in a humidity- and temperature-controlled room in a 12-h light/12-h dark photoperiod (lights on from 07:00–19:00). Photoperiod conditions and experimental manipulations were particularly taken into consideration to maintain reproducibility between experiments. Throughout the duration of the study all animals were handled similarly and supplied with food and water ad libitum.

### 4.2. Experimental Design—Effects of Age and Sex

CP samples of lateral ventricles were dissected from newborn, young (male and female) and adult (male and female) rats after euthanasia under anaesthesia (CO_2_ inhalation) at the same time, between 9 h00 and 11 h00. Effects of age were determined between newborn, young and adult groups and sex effects were evaluated between male and female from the young and adult groups. Since the circulating gonadal hormone concentrations greatly fluctuates in the days that follow birth [18], the effects of sex were not considered in the newborn group.

After collection, CP explants were washed in phosphate-buffered saline and placed immediately in 500 µL of pre-heated complete Dulbecco’s Modified Eagle Medium (DMEM, Life Technologies, Inc., Paisley, UK) with 10% fetal bovine serum (FBS, Biochrom AG, Berlin, Germany) and 1% of penicillin-streptomycin. After 24-h CP explants were collected and frozen in liquid nitrogen for real time RT-PCR analysis. In order to evaluate age and sex-dependent effects in intracellular and secreted protein levels, CP explants and conditioned culture medium from newborn, young (male and female) and adult (male and female) rats were also collected and frozen (−20 °C) for further Western blot analysis.

### 4.3. Experimental Design—Circadian Effects

Intact adult male and female, sham-operated (sham), and ovariectomized (OVX) Wistar Han rats at the age of 2 months ± 2 weeks old were used to study the effect of the circadian rhythm on the expression of Aβ scavengers. Proestrous adult female rats were either bilaterally OVX or sham-operated under general anesthesia, administered as an intraperitoneal injection with a combination of ketamine and medetomidine. Two weeks after surgery, animals were anesthetized again for removal of the brain and CP dissection at specified times. Lights on at 07:00 h was classified as Zeitgeber time (ZT)-0, and sampling times were defined relative to ZT-0. CP tissue was collected from both lateral ventricles at ZT1, ZT7, ZT13, andZT19 (*n* = 4–5 for each time point and each experimental group) and immediately snap frozen in liquid nitrogen as previously described [15,16]. The mRNA expression of ApoJ, Gls, and Ttr was examined by real time RT-PCR.

### 4.4. Real Time RT-PCR

Real time RT-PCR was used to analyse the effects of age, hormonal background and circadian rhythm on the expression of ApoJ, Gls and Ttr genes. Reactions were carried out using 1 µL or 2 µL of cDNA synthesized from 500 ng of total RNA from CP tissues, in a 20 µL reaction mixture containing 10 µL iQ SYBR Green (Thermo Scientific) and 5 ρmol of forward and reverse specific primers. Cyclophilin A (CycA) was used as an endogenous control. Sequences of the primers used are indicated in Table 2. Real time RT-PCR was carried out in a 96-wells plate (Thermo Scientific). The amplification conditions used were 95 °C for 3 min, 40 cycles of 95 °C for 15 s, 58 °C (Apoj, Gls) or 56 °C (Ttr) for 30 s and 72 °C for 30 s. Amplified PCR fragments were checked by melting curves. All primers were previously validated by quantitative PCR reactions with increasing cDNA concentrations, and the reaction efficiencies were calculated. Fluorescence was measured after each cycle and displayed graphically (iCycles iQ Real time detection System Software, Bio-Rad). The software determined the quantification cycle threshold (Ct) values for each sample. Data collected from real time RT-PCR experiments was analysed with the mathematical model proposed by Pfaffl using the formula 2-(ΔΔCt) [41].

### 4.5. Western Blot

Intracellular and secreted APOJ, GLS, and TTR protein levels were also analyzed in order to evaluate age and sex effects. Protein extracts were obtained from frozen CP explants using RIPA lysis buffer (NaCl 150 mM, NP-40 1%, sodium deoxycholate 0.5%, SDS 0.1%, Tris 50 mM) and total protein content was measured using a BCA Protein Assay Kit (Thermo Fisher Scientific, Waltham, MA, USA). Samples containing 25 µg of total protein extracts or 15 µL of culture medium samples were separated by 12.5% SDS-PAGE gels and electrically transferred to polyvinylidene difluoride (PVDF) membranes (Amersham Biosciences). The membranes were blocked for 1 h 30 min with 5% skimmed milk powder in Tris-buffered saline (TBS), and then incubated overnight with rabbit anti-Clusterin/APOJ (0.25 μg/mL, Abcam), rabbit anti-GLS (0.5 µg/mL, Abcam), rabbit anti-TTR antibody (1:250, Dako), or mouse anti-B-actin (1:20,000, Sigma Aldrich). Blots were washed at room temperature with TBS containing 0.1% of Tween (TBS-T) before incubation for 1 h with HRP-conjugated anti-rabbit or anti-mouse secondary antibodies (1:50,000, Santa Cruz Biotechnology, MI, USA). Blots were washed, and antibody binding was detected using the enhanced chemiluminescence assay, ECL substrate (ClarityTM Western ECL Substrate, Bio-Rad, Hercules, CA, USA) according to the manufacturer’s instructions. Images of blots were captured with the ChemiDoc MP Imaging system (Bio-Rad), and densitometry of bands were assessed using the software ImageLab 5.1 (Bio-Rad).

### 4.6. Statistical Analysis

Statistical analysis of the real time RT-PCR and Western blot data was carried out with GraphPad Prism (GraphPad Software Inc., San Diego, CA, USA, Version 7). Data were compared and expressed as mean ± SEM. Comparisons of means were performed using one-way ANOVA followed by the Bonferroni multiple comparison test. Results were considered statistically significant when *p* < 0.05. The CircWave v1.4 analysis software (Dr. Roelof A. Hut) was used to analyze the rhythmicity of ApoJ, Gls, and Ttr expression by a harmonic regression method with an assumed period of 24-h and with alpha set at 0.05.

## Figures and Tables

**Table 1:** Significance (*p*-value) and center of gravity values for each Aβ scavenger’s gene as determined by CircWave analysis.

	Group			
Gene	Intact Females	Intact Males	Sham-Operated	Ovariectomized
*ApoJ*	*p*-value = 0.022 COG = 15.88	NS	*p*-value = 0 COG = 15.3	*p*-value = 0.013 COG = 16.43
*Gls*	NS	NS	NS	NS
*Ttr*	*p*-value = 0.0225 COG = 15.76	*p*-value = 0.0004 COG = 16.15	NS	NS

COG, center of gravity; NS, not significant.

**Table 2:** Sequences of forward and reverse primers used in real time RT-PCR.

Gene	Forward Primer Sequence (5′-3′)	Reverse Primer Sequence (5′-3′)
*Apoj*	CTGACCCAGCAGTACAACGA	AGCTTCACCACCACCTCAGT
*Gls*	GGTGCAGAGGCTCTTCCAGG	CTGCCGGAGCCACACCACTG
*Ttr*	GGACTGATATTTGCGTCTGAAGC	ACTTTCACGGCCACATCGAC
*CycA*	CAAGACTGAGTGGCTGGATGG	GCCCGCAAGTCAAAGAAATTAGAG

## Abbreviations

CP	Choroid plexus
Aβ	Amyloid beta
APOJ	Apolipoprotein J
GLS	Gelsolin
TTR	Transthyretin
CSF	Cerebrospinal fluid
BCSFB	Blood-cerebrospinal fluid barrier
AD	Alzheimer’s disease
